# Characterizing a rare neurogenetic disease, SLC13A5 citrate transporter disorder, utilizing clinical data in a cloud-based medical record collection system

**DOI:** 10.3389/fgene.2023.1109547

**Published:** 2023-03-21

**Authors:** Emily M. Spelbrink, Tanya L. Brown, Elise Brimble, Kirsten A. Blanco, Kimberly L. Nye, Brenda E. Porter

**Affiliations:** ^1^ Stanford University School of Medicine, Department of Neurology and Neurological Sciences, Palo Alto, CA, United States; ^2^ Treatments for Epilepsy and Symptoms of SLC13A5 Foundation, TESS Research Foundation, Menlo Park, CA, United States; ^3^ Invitae, San Francisco, CA, United States; ^4^ Department of Genetics, Stanford University, Stanford, CA, United States

**Keywords:** citrate, transporter, epilepsy, SLC13A5, developmental delay, NACT, rare disease, movement disorder

## Abstract

**Introduction:** SLC13A5 citrate transporter disorder is a rare autosomal recessive genetic disease that has a constellation of neurologic symptoms. To better characterize the neurologic and clinical laboratory phenotype, we utilized patient medical records collected by Ciitizen, an Invitae company, with support from the TESS Research Foundation.

**Methods:** Medical records for 15 patients with a suspected genetic and clinical diagnosis of SLC13A5 citrate transporter disorder were collected by Ciitizen, an Invitae company. Genotype, clinical phenotypes, and laboratory data were extracted and analyzed.

**Results:** The 15 patients reported all had epilepsy and global developmental delay. Patients continued to attain motor milestones, though much later than their typically developing peers. Clinical diagnoses support abnormalities in communication, and low or mixed tone with several movement disorders, including, ataxia and dystonia. Serum citrate was elevated in the 3 patients in whom it was measured; other routine laboratory studies assessing renal, liver and blood function had normal values or no consistent abnormalities. Many electroencephalograms (EEGs) were performed (1 to 35 per patient), and most but not all were abnormal, with slowing and/or epileptiform activity. Fourteen of the patients had one or more brain magnetic resonance imaging (MRI) reports: 7 patients had at least one normal brain MRI, but not with any consistent findings except white matter signal changes.

**Discussion:** These results show that in addition to the epilepsy phenotype, SLC13A5 citrate transporter disorder impacts global development, with marked abnormalities in motor abilities, tone, coordination, and communication skills. Further, utilizing cloud-based medical records allows industry, academic, and patient advocacy group collaboration to provide preliminary characterization of a rare genetic disorder. Additional characterization of the neurologic phenotype will be critical to future study and developing treatment for this and related rare genetic disorders.

## 1 Introduction

Rare diseases, defined as conditions affecting fewer than 200,000 people (‘Orphan Drug Act,’ 1983), are often severe medical conditions that are clinically complex, hard to diagnose, poorly characterized, difficult to treat, and require high levels of healthcare utilization. The term “rare” is both misleading and apt. Although each disease is rare, up to 10,000 individual rare disorders impact, in total, more than 300 million people globally, making rare diseases a major public health problem (Haendel et al., 2020; Tisdale et al., 2021). However, due to the small numbers of patients with each disease, significant challenges exist in the study and development of treatments for rare diseases. These include small numbers, geographically dispersed patients, and the lack of an International Classification of Disease (ICD) code to track and identify patients in healthcare systems. The limited access to disease models and research funding further highlight the need for collaboration between clinicians, industry, and patient advocacy groups to advance rare disease research (Griggs et al., 2009; Merkel et al., 2016; Julkowska et al., 2017; Denton et al., 2021; Denton et al., 2022; Yang et al., 2022).

SLC13A5 citrate transporter disorder is a rare neurological disease caused by pathogenic loss-of-function variants in the *SLC13A5* gene (Thevenon et al., 2014; Hardies et al., 2015; Klotz et al., 2016; Bainbridge et al., 2017; Matricardi et al., 2020; Yang et al., 2020). Despite the initial description 9 years ago (Thevenon et al., 2014), the study of the total disease spectrum has been limited. At present, there exists no comprehensive characterization or prospective metabolic or natural history study for this disorder.

To overcome challenges in characterizing clinical features of the disease, the TESS Research Foundation, serving as a caregiver virtual support network for families impacted by SLC13A5 citrate transporter disorder, asked families to enroll in the Ciitizen platform to allow their medical records to be uploaded to a cloud-based data platform for data collection, extraction, and analysis. Data provided allowed for establishment of baseline laboratory, neurologic, and vital sign findings important for the biomarkers, clinical endpoints, and recognition of safety endpoints for prospective natural history studies and hopefully clinical trials.

The *SLC13A5* gene encodes a sodium-dependent citrate transporter protein, NaCT, that is highly expressed in the liver, brain, bone, teeth, and reproductive organs, and in other organs at much lower levels (Pajor et al., 2001; Inoue, et al., 2002; Gopal et al., 2007; Kumar et al., 2021). Pathogenic variants in NaCT confer an autosomal recessive risk for epilepsy, developmental disability, and a constellation of other phenotypic abnormalities. SLC13A5 was first associated with a pediatric neurologic disorder in 2014 (Thevenon et al., 2014). At that time, the most prominent features of the disorder were recognized, including neonatal onset epilepsy, developmental delay, and prominent abnormal dentition due to amelogenesis imperfecta (Thevenon et al., 2014).

Since its initial description, there has been a significant increase in knowledge about the etiology and clinical manifestations of this monogenic disorder (Hardies et al., 2015; Anselm et al., 2016; Klotz et al., 2016; Bainbridge et al., 2017; Schossig et al., 2017; Weeke et al., 2017; Arvio and Lähdetie, 2020; Matricardi et al., 2020; Yang et al., 2020; Brown et al., 2021; Duan et al., 2021; Goodspeed et al., 2022); although as mentioned previously, a comprehensive characterization phenotypically and metabolically is still lacking. Epilepsy is the most common presentation of SLC13A5 citrate transporter disorder due to its neonatal onset. Almost every patient reported to date presents with seizures in the first hours to days of life (Thevenon et al., 2014; Anselm et al., 2016; Klotz et al., 2016; Matricardi et al., 2020; Yang et al., 2020). Mothers have not consistently reported any difficulties with pregnancy or a history of events during the pregnancy consistent with seizures *in utero* (personnel communication BEP). Infants have normal growth parameters at birth and at least through early childhood (Matricardi et al., 2020; Brown et al., 2021). Brain MRI data are limited, but reports suggest a mixture of transient neonatal and/or persistent white matter abnormalities on T2 and FLAIR sequences (Anselm et al., 2016; Weeke et al., 2017; Whitney et al., 2023). Persistent seizures throughout life have been reported in most patients, and the majority remain on lifelong anti-seizure medications. Rarely, however, patients have been weaned off anti-seizure medications without recurrence of seizures (Matricardi et al., 2020; Yang et al., 2020).

In all patients reported to date, serum, CSF citrate, and methyl citrate levels have been elevated, although only published reports on five patients have had these characterized (Bainbridge et al., 2017). Dental abnormalities are also a consistent finding; small, poorly formed teeth due to amelogenesis imperfecta can have multiple etiologies, but when present, they provide strong diagnostic support for SLC13A5 citrate transporter disorder (Thevenon et al., 2014; Hardies et al., 2015; Klotz et al., 2016; Duan et al., 2021; Dirckx et al., 2022).

Here, we report the clinical diagnosis, developmental history, and basic physical exam findings, including vital signs, laboratory studies, and EEG and MRI results, to expand the characterization of this rare disorder. Although limited in sample size (15 patients), our results demonstrate the ability of the Ciitizen cloud-based method to extract salient clinical features of this rare disease and highlight gaps in present knowledge, allowing for the prediction of clinical care needs. These results also provide baseline data that will be critical for designing future biomarker development studies and treatments.

## 2 Methods

Patients were recruited by the TESS Research Foundation, through internet-based outreach in coordination with a Ciitizen webinar in English to families in the United States. Caregivers of children and adults with a confirmed diagnosis of SLC13A5 citrate transporter disorder based upon clinical symptoms and variants in the *SLC13A5* gene were invited to join the Ciitizen/TESS Research Foundation Databank, which has received an IRB determination of exemption. Ciitizen is a patient-facing platform that collects designated record sets by leveraging the Health Insurance Portability and Accountability Act’s (HIPAA) right of access. To ensure medical record completeness, each participant undergoes a triage process and is required to meet a minimum completeness threshold prior to inclusion in subsequent analyses.

### 2.1 Medical record analysis

Ciitizen has developed a proprietary approach to streamline the generation of regulatory-grade clinical data from unstructured data sources. In brief, medical records are incorporated into the platform for document preprocessing. Through a series of artificial intelligence services, document attributes are determined and verified by human clinicians. To support systematic data capture and harmonization of data sources, Ciitizen extracts information longitudinally from each document in accordance with a standardized data model, encompassing genotype, clinical phenotypes, and therapeutic interventions, among others. Ciitizen has developed a curated ontology that supports the mapping of extracted data to standard codes derived from internationally recognized terminologies, including SNOMED CT, RxNorm, and LOINC. All extracted data were independently verified by two clinicians with relevant training. All genetic variants are reviewed by a certified genetic counselor. Ciitizen data, shared with the TESS Research Foundation, excluded any personally identifying information about the participants or their families. Data used for this publication can be accessed by contacting research@ciitizen.com.

De-identified lab value data from Ciitizen were analyzed using RStudio version 1.4.1106. The code used to analyze the data can be found on GitHub (https://github.com/tanyab37/SLC13A5_patient_deidentified_labs/tree/main). De-identified data points greater than three standard deviations from the normal range or data with missing or incorrect units were excluded from analysis (43 total laboratory data points were excluded).

### 2.2 Developmental milestone analysis

The total number of patients recorded and the specific patients able to achieve each milestone were extracted from the data set. The number of patients unable to achieve the milestone (i.e., specific comment was made that the patient had not achieved the milestone, and the patient never achieved the milestone within available data) is also given. For those able, the average age at which the milestone was first noted and the standard error are included. The ages at which milestones are recorded were limited by examination dates.

## 3 Results

### 3.1 Patient demographics and medical record summary

A total of 15 patients were included in this assessment with an average age of 9.4 ± 5.3 years (median = 9 years) at the time of final data collection (Table 1). The cohort included eight female and seven male patients, each with a clinical diagnosis of SLC13A5 citrate transporter disorder confirmed via a clinical history and genetic report with variants in the *SLC13A5* gene. Since this disease was identified in 2014 (Thevenon et al., 2014), there has been a paucity of functional data associated with the large number of SLC13A5 variants reported, which is an important component used to determine ACMG classification (Richards et al., 2015). All variants were reviewed by a certified genetic counselor and clinicians (Table 1). One patient, patient ID 4, did not have genetic testing reported. Patient ID 4 has a clinical history consistent with SLC13A5 citrate transporter disorder and a biological sibling, ID 7, with a genetic report. Thus, we have included this patient in the analyses. Although there are reports in the scientific literature of SLC13A5 citrate transporter disorder patients in their fifties (Arvio and Lähdetie, 2020), the oldest patient in this cohort was 18 years at the last recorded data point.

**TABLE 1:** Patient demographics. SLC13A5 citrate transporter disorder patients included in this assessment are listed by Patient ID, age at the time of publication, age at diagnosis, sex, SLC13A5 variant, and current ACMG classification. Variants were assessed by a certified genetic counselor; NM_177550.5 NP808218.1

Patient ID	Age at enrollment (y)	Age at diagnosis (y)	Sex	*SLC13A5* variant	NaCT variant	ACMGClassification
1	1.8	0.2	F	c.650G>A	p.S217N	VUS
				c.1599C>A	p.N533K	VUS
2^a^	12.1	4.5	F	c.245A>G	p.Y82C	VUS
				c.655G>A	p.G219R	P
3^b^	15.8	8.1	F	c.511delG	p.E171fs	P
				c.511delG	p.E171fs	P
4* ^c^	9.0		F			
5	8.3	6.3	M	c.997C>T	p.R333*	P
				c.997C>T	p.R333*	P
6	2.8	0.4	M	c.1460C>T	p.P487L	VUS
				c.232–2A>G		LP
7^c^	13.3	5.5	F	c.368 + 1G>A		LP
				c.368 + 1G>A		LP
8^d^	18.1	10.6	F	c.1475T>C	P.492P	LP
				c.655G>A	P.219R	P
9	9.5	4.4	M	c.1511delT	P.504fs	LP
				c.1511delT	P.504fs	LP
10^b^	14.7	6.8	M	c.511delG	P.171fs	P
				c.511delG	P.171fs	P
11^d^	8.4	0.9	M	c.1475T>C	P.492P	LP
				c.655G>A	P.219R	P
12	4.1	0.2	F	c.425C>T	P.142M	LP
				Deletion		P
13	5.7	0.1	F	c.389G>A	P.130D	VUS
				Deletion		P
14	2.9	0.1	M	c.997C>T	P.333*	P
				c.1514C>T	P.505L	VUS
15^a^	15.0	7.1	M	c.245A>G	P.82C	VUS
				c.655G>A	P.219R	P

**^For Patient ID:^** Genetic testing results are unavailable, and ^a,b,c,d^ indicate biological siblings.

P, pathogenic; LP, likely pathogenic; VUS, variant of uncertain significance.

The medical records extracted for this article include 8077 pages of medical records, with a mean of 538.5 ± 266.4 pages reviewed per patient. This includes medical records from 80 institutions and the sum of 115 patient years analyzed.

### 3.2 Developmental milestones

**FIGURE 1:**
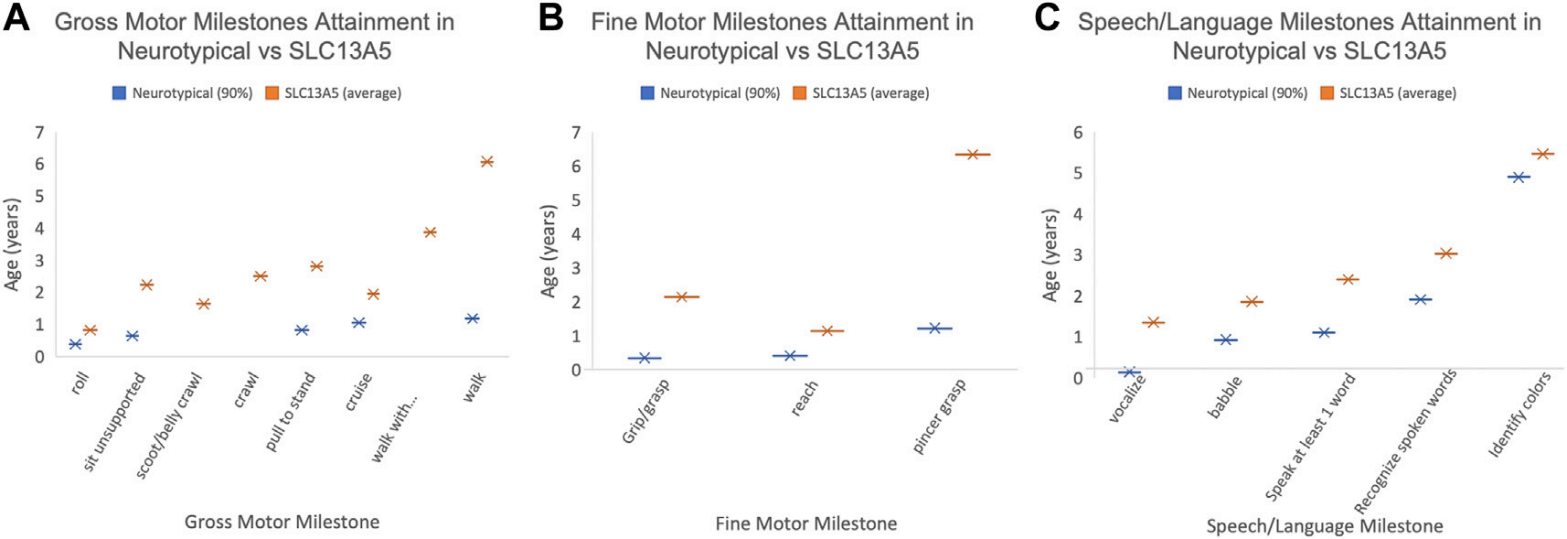
Patient developmental milestones. The 90th percentile for milestone attainment in neurotypical patients (blue, as defined by the Denver Developmental Milestone Screening (Frankenburg and Dodds, 1967) compared to the average milestone attainment age for the SLC13A5 citrate transporter disorder patients. Only those patients with documentation to support the timing of milestone attainment are included. **(A)** Gross motor milestones. **(B)** Fine motor milestones. **(C)** Speech/language milestones. Only patients with documented milestones were used to calculate the average age.

To determine the time course of neurodevelopment milestone attainment, we assessed the age at which patients achieved specific gross and fine motor skills, and speech and language and academic skills (Table 2). Not all patients had a documentation of gross motor skills in their medical records. Of the patients with gross motor skills documented, most eventually achieved early developmental milestones. Patients were able to roll on average at 0.83 years (10 months), compared to 0.39 years (4.7 months), the 90th percentile for neurotypical children (Frankenburg and Dodds, 1967). Gross motor skills requiring greater whole-body coordination and tone, such as sitting without support and pulling to stand, were achieved later in the SLC13A5 citrate transporter disorder patients than typically developing children (Figure 1; Table 2). The ability to walk is a significant developmental skill with good documentation of impact on the patient quality of life. Although a majority (nine) of SLC13A5 citrate transporter disorder patients eventually gained the ability to walk with or without assistance, this was attained much later than that in neurotypical children. Nine patients were able to walk with assistance on average at 3.9 years, and four patients were able to walk independently starting at an average of 6.1 years old. Interestingly, of the two patients assessed for their ability to jump, neither (at 15 and 18 years old, respectively) had the ability, suggesting sustained problems with motor coordination. Four patients had documented periods of gross motor regressions at ages ranging from 0.5 to 10.2 years. Additionally, 10 patients reported using wheelchairs, walkers, or strollers to assist with impaired mobility.

**TABLE 2:** Developmental milestone achievement and assistive device use. Summary description of developmental milestone achievement and the assistive device use for patients for whom these data were available in this dataset. Developmental milestones: The total number of patients recorded as able and which patients were able to achieve each milestone are listed. The number of patients who were unable to achieve milestones (i.e., a specific comment was made that the patient had not achieved the milestone, and the patient never achieved the milestone within available data) is also given. For those noted as able, the average age at which the milestone was first noted and the standard error are included, as well as sex. Assistive device use: Total number and specific patients using assistive devices (motor or communication) are noted.

Developmental Milestones	# Able	Age Able (years)	SE	Sex F/M	# Unable
Gross Motor					
Control head posture	4/15 (4, 6, 9, and 11)	0.64	0.49	1/3	3/15 (2, 5, and 15)
Roll	12/15 (1, 2, 3, 4, 5, 6, 8, 9, 10, 11, 13, and 14)	0.83	0.23	6/6	1/15 (7)
Siting unsupported	5/15 (3, 4, 5, 13, and 15)	2.24	1.00	3/2	4/14 (2, 6, 9, and 14)
Scoot/belly crawl	3/15 (11, 12, and 14)	1.65	0.57	1/2	0/15
Crawl	7/15 (3, 4, 7, 10, 11, 13, and 15)	2.51	0.73	4/3	4/15 (2, 5, 6, and 9)
Pull to stand	6/15 (3, 4, 7, 8, 10, and 15)	2.81	0.89	4/2	0/15
Cruise	4/15 (3, 4, 7, and 11)	1.96	0.25	2/1	0/15
Walk with assistance	9/15 (3, 4, 5, 7, 9, 10, 11, 13, and 15)	3.88	0.84	4/5	0/15
Walk	4/15 (4, 8, 10, and 11)	6.08	2.69	2/2	
Jump	0/15				2/15 (7 and 8)
Gross motor regression	4/15 (2, 3, 9, and 10)	3.68	2.55	2/2	
Fine Motor					
Grip/grasp	7/15 (3, 6, 9, 10, 11, 13, and 14)	2.14	0.89	2/5	1/15 (2)
Reach	9/15 (1, 3, 4, 6, 7, 9, 11, 13, and 14)	1.14	0.42	5/4	2/15 (2 and 5)
Pincer grasp	4/15 (3, 4, 8, and 10)	6.34	2.15	3/1	2/15 (5 and 13)
Write	1/15 (8)	10.94	NA	1/0	1/15 (13)
Speech/Language					
Communicate non-verbally	6/15 (3, 7, 12, 13, 14, and 15)	3.64	1.21	4/2	0/15
Vocalize	8/15 (1, 2, 3, 4, 5, 6, 7, and 9)	1.36	0.53	5/3	0/15
Babble	10/15 (1, 2, 3, 4, 5, 7, 9, 11, 13, and 15)	1.87	0.57	6/4	0/15
Use sign language	2/15 (3 and 11)	4.27	3.96	1/1	2/15
Speak at least one word	8/15 (3, 4, 7, 8, 9, 10, 11, and 14)	2.41	0.53	6/4	4/15 (2, 5, 12, 15)
Recognize spoken words	6/15 (3, 5, 7, 8, 11, and 13)	3.04	0.69	4/2	0/15
Language regression	1/15 (3)	1.59		1/0	
Academic					
Identify colors	4/15 (3, 7, 11, and 15)	5.46	0.48	2/2	0/15
Count to 10	1/15 (8)	10.98	NA	1/0	0/15
Recognize letters/numbers/symbols	4/15 (3, 7, 8, and 11)	6.64	1.04	3/1	0/15
Read	2/15 (7 and 8)	9.36	1.65	2/0	0/15
Write	1/15 (8)	10.94	NA	2/0	0/15
Augmentive Devices	# using				
Motor augmentive	10/15 (2, 3, 5, 7, 9, 10, 11, 13, 14, and 15)			4/6	
Alternate augmentative communication	7/15 (5, 7, 9, 10, 11, 13, and 15)			2/5	

Another common feature of SLC13A5 citrate transporter disorder is intellectual disability. To begin to understand intellectual skill development, we assessed patients for language and academic abilities. Ten patients were able to babble beginning on average at 1.9 years of age, and eight patients could speak at least one word on average at 2.4 years of age. Six patients could recognize spoken words at 3 years old. Seven patients reported using a communication assistance device (Figure 1; Table 2). Though multiple patients demonstrated the ability to communicate and many needed communication aids, only patient showed language regression at 1.6 years. Spanning both academic and language abilities, four patients were able to identify colors starting at 5.5 years old and four patients could recognize letters/numbers/symbols starting at 6.6 years old. Two patients were documented as being able to read starting at 9.4 years old, and one patient was documented as being able to write beginning at 10.9 years old. Altogether, SLC13A5 citrate transporter disorder patients demonstrate modest ability to communicate, often facilitated by communication assistance devices. Patients also appear to slowly develop new skills throughout childhood, with some periods of language or motor regression in a few patients.

### 3.3 Clinical diagnosis and exam findings

The records were assessed for evidence of clinical diagnosis; all had a diagnosis of epilepsy (Table 3). Four patients had a diagnosis of epileptic encephalopathy; see below Section 3.4 and Table 4 for discussion on EEG findings. All patients had developmental delay, with eight patients having some version of language disorder noted. Three patients had noted developmental regression. Weakness or low tone were noted in all but two of the patients; however, four of these patients with low tone or weakness also had spasticity documented. Movement disorders of various types were recorded, with six patients having ataxia, dystonia, and/or choreoathetosis.

**TABLE 3:** Clinical diagnosis summary. Clinical diagnoses extracted from the medical records were subdivided into three categories: epilepsy, neurodevelopmental delay, and movement.

Diagnosis	Patients
Epilepsy	
Epilepsy	All patients
Epileptic encephalopathy	3, 7, 14, and 15
Neurodevelopment	
Global developmental delay	All patients
Developmental regression	2, 3, and 10
Altered mental status	3
Expressive language disorder	2, 4, and 11
Mixed receptive-expressive language disorder	5, 8, 9, and 14
Receptive language disorder	10
Movement	
Muscle weakness	2, 4, 9, 10, 11, and 15
Hemiplegia	11 and 14
Axial hypotonia	10
Mixed muscle tone	3, 10, and 15
Decreased muscle tone	4, 5,7, 8, 9, 11, 12, 13, and 14
Appendicular hypertonia	10
Facial hypotonia	10
Spasticity	3, 7, 9, and 10
Torticollis	1,6,11
Ataxic cerebral palsy	15
Ataxia	4, 7, 8, 10, and 11
Dyskinesia	3
Hyperkinesia	15
Incoordination	3
Dystonia	3, 4, 7, 8, 10, and 11
Choreoathetosis	3, 7, 10, and 15
Abnormal gait	3

**FIGURE 2:**
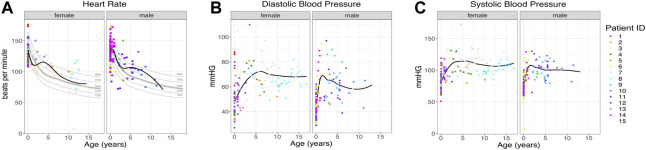
Patient vital signs. **(A)** Heart rate per patient. The black line indicates the trend line. Gray lines indicate the 10th, 25th, 50th, 75th, and 90th percentiles by age (Fleming et al., 2011), *n* = 259. **(B, C)** Diastolic and systolic blood pressure for each patient, *n* = 700. The black line indicates the trend line. Individual patients are indicated by individual colors.

Vital signs, heart rate, and blood pressure had wide value ranges, as would be expected for information collected during outpatient and inpatient visits. The total number of vital signs measured was large (total of 959 vital signs, average of 64 per patient, range: 14–126 per patient). Overall, vital signs mostly fell in the normal range with no persistent trends in either direction. (Figure 2).

### 3.4 Laboratory, EEG, and MRI results

**FIGURE 3:**
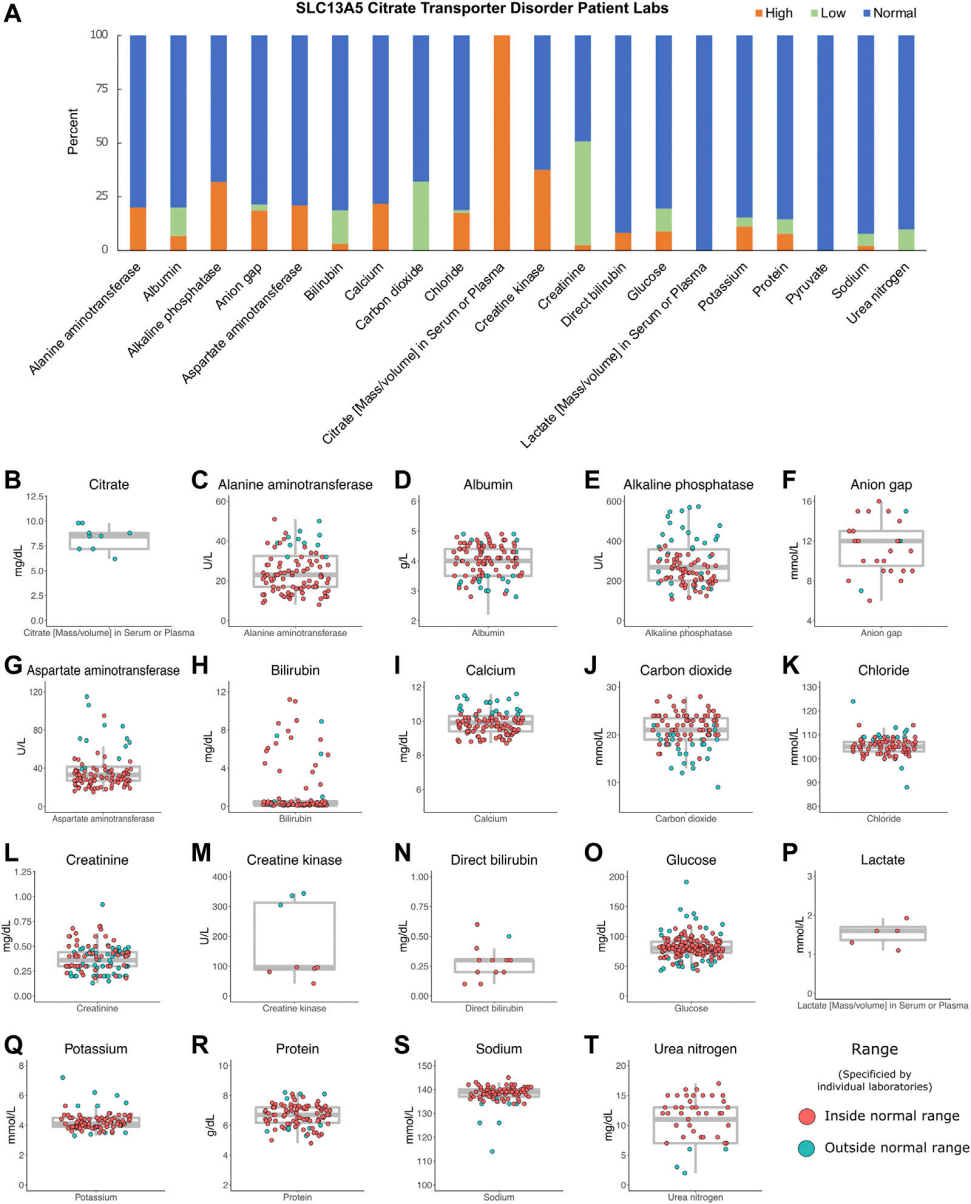
Patient labs. **(A)** Percentage of patient labs as high, normal, or low. Evaluation of high, normal, and low values was determined for each individual laboratory assessed and compared to reference levels provided by each institution. **(B–T)** Individual labs by patient, *n* = 2,251. Gray box indicates the median, interquartile ranges, and minimum and maximum values without outliers. The color indicates labs inside or outside the normal range, as determined by individual laboratory reports.

To establish range and trends for common laboratory studies from the patients, records were assessed for basic and metabolic laboratory studies. Patients had 2,251 laboratory studies performed, with an average of 150 laboratory studies per patient (range: 16–326) as part of the ongoing clinical care. These included complete blood counts, electrolytes, and renal and liver function studies. Overall, they varied around the normal range with no trends in either direction except for serum citrate, which was several fold elevated in all three patients studied (Figure 3).

All the patients had at least one EEG result report, and the majority had many more, ranging 1–35 per patient (Table 4). This is in accordance with the common diagnosis of epilepsy. Although 10 patients had at least one normal EEG (range: 0–5), most were abnormal, but notably, the majority of EEGs for each patient were not described as epileptiform; instead, slowing or other non-epileptiform features were reported.

**TABLE 4:** EEG report summary. Total number of normal, abnormal, and epileptiform EEGs are listed for each patient.

Patient #	# Normal EEG	# Abnormal EEG	# Epileptiform EEG
1	1	2	1
2	0	22	9
3	3	19	10
4	1	3	2
5	2	18	13
6	0	16	15
7	3	14	11
8	0	18	17
9	1	0	0
10	2	3	1
11	5	17	10
12	1	12	6
13	1	8	7
14	0	7	4
15	0	5	5

To assess brain structure, the medical records were reviewed for brain imaging studies. Fourteen of the patients had at least one brain MRI reported (Table 5). Three patients had only a normal MRI report, and another four patients had at least one normal brain MRI report. Interestingly, only four patients had findings that were previously reported in SLC13A5 citrate transporter disorder patients, consistent with the finding that punctate white matter lesions occur in a subset of cases (Weeke et al., 2017; Whitney et al., 2023). The other abnormalities found in this cohort were only present in a single patient and unlikely to have contributed to the complex neurologic phenotype except possibly the patient with bilateral polymicrogyria.

**TABLE 5:** MRI report summary. All MRI reports extracted from the medical records and the number of normal and abnormal scans with short description of each abnormal result is listed for each patient.

Patient	Normal brain MRI scans	Abnormal brain MRI scans	Abnormality
1	1	0	
2	0	3	Cerebral edema
			Asymmetry of the cerebral hemispheres
			Status post-procedure and cortical dysplasia
3	2	4	Acute infarct
			Periventricular white matter T2 hyperintensity
			Hypoplasia of the corpus callosum and nodular heterotopia
4	0	1	Acute infarct
5	1	4	Trigonocephaly and subdural hematoma
			Subacute hemorrhage
			Trigonocephaly, subdural hematoma, and simple craniosynostosis
6	0	5	Thrombosis of superior sagittal sinus
			Thrombosis of superior sagittal sinus
			Disorder of neuronal migration and differentiation and thrombosis of superior sagittal sinus
			Abnormal intracranial vein
			Cerebral cyst
7	0	2	Abnormal restricted diffusion and gliosis
8	0	3	Right mesial temporal lobe sclerosis
			Right mesial temporal lobe sclerosis
			Right mesial temporal lobe sclerosis
9	0	1	Periventricular white matter T2 hyperintensity, confluent, and cerebral ventriculomegaly
10	1	2	Periventricular white matter FLAIR hyperintensity;
			Abnormal restricted diffusion, cerebral cyst, and signal abnormality of the midbrain
11	1	0	
12	1	0	
13	0	0	
14	0	1	Bilateral polymicrogyria
15	1	1	Gliosis, right frontal lobe, status post-procedure, subcortical T2 hyperintensity, T2 hyperintensity of the thalamus, and hypertrophy of adenoids

T2, transverse relaxation time; FLAIR, fluid-attenuated inversion recovery.

## 4 Discussion

Our findings from the medical records of the 15 patients with SLC13A5 citrate transporter disorder support and expand the current body of knowledge about the phenotype of this rare disorder and exemplify the utility and limitations to this cloud-based record data for studying a rare disease.

We confirm that epilepsy is a pervasive symptom in SLC13A5 citrate transporter disorder (Thevenon et al., 2014; Hardies et al., 2015; Klotz et al., 2016; Schossig et al., 2017; Matricardi et al., 2020; Yang et al., 2020). We further expand on prior reports of developmental delay and motor exam abnormalities, including data from clinical diagnosis, assistive equipment, and attainment of specific motor and cognitive development skills. Overall, SLC13A5 citrate transporter disorder patients continue to develop new skills but with profound delays compared to typically developing children. Our data show significantly delayed attainment of motor milestones. For example, the ability to sit unsupported was first reported in five patients at 2.2 years on average. Unassisted walking occurred in four patients at 6.1 years on average (Figure 1). It should be noted that the ages at which milestones are recorded are limited by the age examined. The consistent trend is for persistent gross motor delays and disability but with ability for slow forward progress in motor domains.

Rare regression or loss of skills was noted—in motor milestones for four patients and language skills for one patient. This was reported at a single time point, and only 3/15 had a developmental regression clinical diagnosis recorded. Patient 3 had both language and motor regression, both noted at the 1.6y encounter. There was not a consistent age at which regression, when present, occurred (0.5–10.2 years) (Table 2).

Overall, these data demonstrate a lack of motor or language regression in SCL13A5 citrate transporter disorder, but persistent global developmental delays and impaired gross motor coordination. This conclusion is concordant with the frequent diagnosis coding of hypotonia/mixed tone, disordered language, and incoordination or disordered movement (Table 3). However, this dataset includes at times contradictory findings or diagnoses on serial encounters for the same patient. For example, while low tone or muscle weakness was noted in many, a few had mixed tone or even a spasticity diagnosis. Movement disorder was variably characterized as ataxia, dyskinesia, hyperkinesia, dystonia, myoclonus, and chorea/choreoathetosis. Communication disorders—expressive, receptive, and mixed—were common, as was the use of augmentative communication devices. A more extensive movement and coordination battery will be important for patient assessment, and how to measure most effectively deficits and changes over time for these patients.

We evaluated hundreds of laboratory results and vital signs for SLC13A5 citrate transporter disorder patients to establish baseline values for this disorder. Medical records contained many assessments of common laboratory studies. Outside of a several-fold elevation in plasma citrate, other laboratory findings, including those assessing renal and liver function, were normal (Figure 3). SLC13A5 expression is highest in the liver, but interestingly, transaminase and serum protein values were mostly in the normal range. Our evaluation found a normal heart rate and blood pressure range for age (Figure 2). Although individual patients intermittently had elevated heart rates (Figure 2) or an isolated laboratory value out of range (Figure 3), there was no consistent elevation or depression found. Interestingly, the present data differ from SLC13A5-knockout rodent models that show reduced blood pressure and heart rates (Willmes et al., 2021). Furthermore, pediatric patients included here had a high number of vital measurements (Figure 2), likely secondary to the increased healthcare system utilization. The associated “white coat” stress can be associated with an elevated heart rate and blood pressure in a medical setting (Franklin et al., 2013). The robust overall maintenance of normal vital signs, despite the challenges of ongoing seizures, medications, and hospital settings, suggests minimal systemic effects of the disorder.

Citrate levels were elevated in the sera of the three SLC13A5 citrate transporter disorder patients tested (Figure 3), confirming the research metabolomics data previously reported (Bainbridge et al., 2017). This adds to the body of data on five patients with elevated citrate levels previously published (Bainbridge et al., 2017). Interestingly, SLC13A5−/− rodent models and *in vitro* models harboring patient mutations in the *SLC13A5* gene all lacked citrate transport and reported elevated citrate levels (Hardies et al., 2015; Klotz et al., 2016; Pajor et al., 2016; Selch et al., 2018; Henke et al., 2020; Kumar et al., 2021; Sauer et al., 2021; Dirckx et al., 2022). The data highlight that serum citrate level elevation might be used as a biomarker for the disorder in both mice and humans. However, the causative role of increased extracellular citrate increased has not been explored. Together, these data suggest that serum citrate may provide a useful biomarker for SLC13A5 citrate transporter disorder. Future studies are needed to determine whether serum citrate levels correlate with disease severity.

The EEG findings for SLC13A5 are not those of a severe epileptic encephalopathy, in which frequent or abundant interictal spikes are a dominant feature of the EEG and suspected as primary drivers of developmental, cognitive, and behavioral pathology (Berg et al., 2010; Scheffer and Liao, 2020). Most of the EEGs were either normal or had non-epileptiform abnormalities such as slowing of the background (although this varied considerably by patient)—consistent with developmental delays and encephalopathy but not epileptic encephalopathy. The rest of the studies did have epileptiform activity, either with sharp transients, spikes, or seizures captured. These findings suggest SLC13A5 citrate transporter disorder is a developmental and epileptic encephalopathy (DEE) (Stafstrom and Kossoff, 2016; Scheffer and Liao, 2020), in which biological processes caused by the disorder—both related to and independent of seizures and epilepsy—impact development, and the interplay of both might determine cognitive and developmental outcomes.

The brain MRI findings were in overall agreement with previous findings. Brain MRIs in our dataset were normal in four patients or had T2 or FLAIR hyperintensities in the white matter in four other patients. Remaining patients did not have the FLAIR abnormalities noted and had a unique finding not consistent across the remaining patients (Table 5). A mesial temporal lesion in the right hippocampus in Patient 8 could be related to a prolonged bout of status epilepticus. Overall, this suggests grossly normal brain morphology and mild changes to myelinated regions of the brain in a subset of patients, supported by a recent publication, with 66% of MRIs being normal and white matter changes the most common abnormality (Whitney et al., 2023). We included subjects who could possibly have dual diagnoses, although none were known to have a second etiology for their disorder, e.g., polymicrogyria, acute infarcts, sinus thromboses, and hippocampal sclerosis. It is unclear whether at least some of these may be sequelae to SLC13A5 citrate transporter disorder and frequent seizures vs. superimposed primary insults or incidental findings.

We show that cloud-based medical record collection and evaluation is effective at initial characterization of rare diseases. It establishes essential baseline laboratory and vital sign data for future clinical trials. It helps highlight phenotypic features that will require careful characterization in a natural history study, for example, tone and movement disorder in SLC13A5 citrate transporter disorder, which frequently had conflicting documentation within a single subject on sequential exams, as noted previously. It also highlights prominently lacking data—for example, despite the name citrate transporter disorder, only 3 of the 15 patients had blood citrate levels documented in medical records. The data presented support the utility of common data elements and milestone checklists at clinical appointments to improve utilization of medical records for advancing research in rare neurodevelopmental disorders.

There is significant selection bias possible in terms of who enrolls in the Ciitizen database. Those with more severe disease may be more likely to have been diagnosed and more motivated to enroll; conversely, few older patients are enrolled (possibly due to the disorder being more recently recognized), and families of deceased patients did not enroll (possibly skewing data away from fatal cases). Outreach via webinar and social media was in English, and this, along with a lack of access to testing and a lack of medical record compatibility, limited the diversity and international scope of the analysis. A study on how to best overcome these limitations may be crucial to improving research and equitably understanding rare diseases.

These data lay the foundation for future studies by providing detailed phenotypic information in measures beyond seizures, establishing general SLC13A5 citrate transporter disorder normative laboratory values for future clinical trials, and beginning to characterize motor and cognitive development in SLC13A5 citrate transporter disorder patients. Finally, this project models a successful partnership amongst academics, industry, and patient advocacy groups to advance patient-centered, rare disease research. We hope that similar partnerships may provide effective foundations for the launch of better-designed research into rare diseases.

## Data Availability

These data can be accessed at: contacting research@ciitizen.com and (https://github.com/tanyab37/SLC13A5_patient_deidentified_labs/tree/main).

## References

[B1] Anselm I. MacCuaig M. Prabhu S. B. Berry G. T. (2016). Disease heterogeneity in Na+/Citrate cotransporter deficiency. JIMD Rep. 31, 107–111. 10.1007/8904_2016_546 26960556 PMC5388636

[B2] Arvio M. Lähdetie J. (2020) ‘Adult phenotype of the homozygous missense mutation c.655G>A, p.Gly219Arg in SLC13A5: A case report, Am. J. Med. Genet. Part A, 182(11), 2671–2674. 10.1002/ajmg.a.61802 33200910

[B3] Bainbridge M. N. Cooney E. Miller M. Kennedy A. D. Wulff J. E. Donti T. (2017). Analyses of SLC13A5-epilepsy patients reveal perturbations of TCA cycle. Mol. Genet. metabolism 121 (4), 314–319. 10.1016/j.ymgme.2017.06.009 PMC7539367 28673551

[B42] Berg A. T. Berkovic S. F. Brodie M. J. Buchhalter J. Cross J. H. van Emde Boas W. (2010). Revised terminology and concepts for organization of seizures and epilepsies: Report of the ILAE Commission on Classification and Terminology. Epilepsia 51 (4), 676–685. 10.1111/j.1528-1167.2010.02522.x 20196795

[B4] Brown T. L. Nye K. L. Porter B. E. (2021). Growth and overall health of patients with SLC13A5 citrate transporter disorder. Metabolites 11 (11), 746. 10.3390/metabo11110746 34822404 PMC8625967

[B5] Cherian C. Appendino J. P. Ashtiani S. Federico P. Molnar C. P. Kerr M. (2022). The phenotypic spectrum of KCNT1: A new family with variable epilepsy syndromes including mild focal epilepsy. J. Neurology 269 (4), 2162–2171. 10.1007/s00415-021-10808-y 34537872

[B6] Denton N. Molloy M. Charleston S. Lipset C. Hirsch J. Mulberg A. E. (2021). Data silos are undermining drug development and failing rare disease patients. Orphanet J. Rare Dis. 16 (1), 161. 10.1186/s13023-021-01806-4 33827602 PMC8025897

[B7] Denton N. Mulberg A. E. Molloy M. Charleston S. Fajgenbaum D. C. Marsh E. D. (2022). Sharing is caring: A call for a new era of rare disease research and development. Orphanet J. Rare Dis. 17 (1), 389. 10.1186/s13023-022-02529-w 36303170 PMC9612604

[B8] Dirckx N. Zhang Q. Chu E. Y. Tower R. J. Guo S. Park A. (2022). A specialized metabolic pathway partitions citrate in hydroxyapatite to impact mineralization of bones and teeth. Proc. Natl. Acad. Sci. 119 (45), e2212178119. 10.1073/pnas.2212178119 36322718 PMC9659386

[B9] Duan R. Saadi N. W. Grochowski C. M. Bhadila G. Faridoun A. Mitani T. (2021). A novel homozygous SLC13A5 whole-gene deletion generated by Alu/Alu-mediated rearrangement in an Iraqi family with epileptic encephalopathy. Am. J. Med. Genet. 10.1002/ajmg.a.62192 PMC8445493 33797191

[B10] Fleming S. Thompson M. Stevens R. Heneghan C. Pluddemann A. Maconochie I. (2011). Normal ranges of heart rate and respiratory rate in children from birth to 18 years of age: A systematic review of observational studies. Lancet 377 (9770), 1011–1018. 10.1016/S0140-6736(10)62226-X 21411136 PMC3789232

[B11] Frankenburg W. K. Dodds J. B. (1967). The denver developmental screening test. J. Pediatr. 71 (2), 181–191. 10.1016/S0022-3476(67)80070-2 6029467

[B12] Franklin S. S. Thijs L. Hansen T. W. O'Brien E. Staessen J. A. (2013). White-coat hypertension: New insights from recent studies. Hypertension 62 (6), 982–987. 10.1161/HYPERTENSIONAHA.113.01275 24041952

[B13] Goodspeed K. Liu J. S. Nye K. L. Prasad S. Sadhu C. Tavakkoli F. (2022). SLC13A5 deficiency disorder: From genetics to gene therapy. Genes 13 (9), 1655. 10.3390/genes13091655 36140822 PMC9498415

[B14] Gopal E. Miyauchi S. Martin P. M. Ananth S. Srinivas S. R. Smith S. B. (2007) ‘Expression and functional features of NaCT, a sodium-coupled citrate transporter, in human and rat livers and cell lines’. Am. J. Physiology-Gastrointestinal Liver Physiology, 292(1), G402–G408. 10.1152/ajpgi.00371.2006 16973915

[B15] Griggs R. C. Batshaw M. Dunkle M. Gopal-Srivastava R. Kaye E. Krischer J. (2009). Clinical research for rare disease: Opportunities, challenges, and solutions. Mol. Genet. Metabolism 96 (1), 20–26. 10.1016/j.ymgme.2008.10.003 PMC3134795 19013090

[B16] Haendel M. Vasilevsky N. Unni D. Bologa C. Harris N. Rehm H. (2020). How many rare diseases are there? Nat. Rev. Drug Discov. 19 (2), 77–78. 10.1038/d41573-019-00180-y 32020066 PMC7771654

[B17] Hardies K. de Kovel C. G. F. Weckhuysen S. Asselbergh B. Geuens T. Deconinck T. (2015). Recessive mutations in SLC13A5 result in a loss of citrate transport and cause neonatal epilepsy, developmental delay and teeth hypoplasia. Brain A J. Neurology 138, 3238–3250. 10.1093/brain/awv263 26384929

[B18] Henke C. Tollner K. van Dijk R. M. Miljanovic N. Cordes T. Twele F. (2020). Disruption of the sodium-dependent citrate transporter SLC13A5 in mice causes alterations in brain citrate levels and neuronal network excitability in the hippocampus. Neurobiol. Dis. 143, 105018. 10.1016/j.nbd.2020.105018 32682952

[B19] Inoue K. Zhuang L. Ganapathy V. (2002). Human Na+-coupled citrate transporter: Primary structure, genomic organization, and transport function. Biochem. Biophysical Res. Commun. 299 (3), 465–471. 10.1016/S0006-291X(02)02669-4 12445824

[B20] Julkowska D. Austin C. P. Cutillo C. M. Gancberg D. Hager C. Halftermeyer J. (2017). The importance of international collaboration for rare diseases research: A European perspective. Gene Ther. 24 (9), 562–571. 10.1038/gt.2017.29 28440796 PMC5628265

[B21] Klotz J. Porter B. E. Colas C. Schlessinger A. Pajor A. M. (2016). Mutations in the Na+/Citrate cotransporter NaCT (SLC13A5) in pediatric patients with epilepsy and developmental delay. Mol. Med. 22, 310–321. 10.2119/molmed.2016.00077 27261973 PMC5023510

[B22] Kumar A. Cordes T. Thalacker-Mercer A. E. Pajor A. M. Murphy A. N. Metallo C. M. (2021). *NaCT (*SLC13A5*) facilitates citrate import and metabolism under nutrient-limited conditions* . Cell Rep. 36: 109701. 10.1101/2021.04.08.439058 34525352 PMC8500708

[B23] Matricardi S. De Liso P. Freri E. Costa P. Castellotti B. Magri S. (2020). Neonatal developmental and epileptic encephalopathy due to autosomal recessive variants in *SLC13A5* gene. Epilepsia 61 (11), 2474–2485. 10.1111/epi.16699 33063863

[B24] Merkel P. A. Manion M. Gopal-Srivastava R. Groft S. Jinnah H. A. Robertson D. (2016). The partnership of patient advocacy groups and clinical investigators in the rare diseases clinical research network. Orphanet J. Rare Dis. 11, 66. 10.1186/s13023-016-0445-8 27194034 PMC4870759

[B25] ‘Orphan Drug Act’ (1983). Pub L. No. 97-414, 96 Stat.2049.

[B26] Pajor A. M. de Oliveira C. A. Song K. Huard K. Shanmugasundaram V. Erion D. M. (2016). Molecular basis for inhibition of the Na ^+^/citrate transporter NaCT (SLC13A5) by dicarboxylate inhibitors. Mol. Pharmacol. 90 (6), 755–765. 10.1124/mol.116.105049 27683012

[B27] Pajor A. M. Gangula R. Yao X. (2001). Cloning and functional characterization of a high-affinity Na+/dicarboxylate cotransporter from mouse brain. Am. J. Physiology-Cell Physiology 280 (5), C1215–C1223. 10.1152/ajpcell.2001.280.5.C1215 11287335

[B28] Reynolds C. King M. D. Gorman K. M. (2020). The phenotypic spectrum of SCN2A-related epilepsy. Eur. J. Paediatr. Neurology 24, 117–122. 10.1016/j.ejpn.2019.12.016 31924505

[B29] Richards S. Aziz N. Bale S. Bick D. Das S. Gastier-Foster J. (2015). Standards and guidelines for the interpretation of sequence variants: A joint consensus recommendation of the American College of medical genetics and genomics and the association for molecular pathology. Genet. Med. 17 (5), 405–424. 10.1038/gim.2015.30 25741868 PMC4544753

[B30] Sauer D. B. Song J. Wang B. Hilton J. K. Karpowich N. K. Mindell J. A. (2021). Structure and inhibition mechanism of the human citrate transporter NaCT. Nature 591 (7848), 157–161. 10.1038/s41586-021-03230-x 33597751 PMC7933130

[B31] Scheffer I. E. Liao J. (2020). Deciphering the concepts behind “Epileptic encephalopathy” and “Developmental and epileptic encephalopathy”. Eur. J. Paediatr. Neurology 24, 11–14. 10.1016/j.ejpn.2019.12.023 31926847

[B32] Schossig A. Bloch-Zupan A. Lussi A. Wolf N. I. Raskin S. Cohen M. (2017). SLC13A5 is the second gene associated with Kohlschütter–Tönz syndrome. J. Med. Genet. 54 (1), 54–62. 10.1136/jmedgenet-2016-103988 27600704

[B33] Selch S. Chafai A. Sticht H. Birkenfeld A. L. Fromm M. F. Konig J. (2018). Analysis of naturally occurring mutations in the human uptake transporter NaCT important for bone and brain development and energy metabolism. Sci. Rep. 8, 11330. 10.1038/s41598-018-29547-8 30054523 PMC6063891

[B34] Stafstrom C. E. Kossoff E. H. (2016). Epileptic encephalopathy in infants and children. Epilepsy Curr. 16 (4), 273–279. 10.5698/1535-7511-16.4.273 27582673 PMC4988066

[B35] Thevenon J. Milh M. Feillet F. St-Onge J. Duffourd Y. Juge C. (2014). Mutations in SLC13A5 cause autosomal-recessive epileptic encephalopathy with seizure onset in the first days of life. Am. J. Hum. Genet. 95 (1), 113–120. 10.1016/j.ajhg.2014.06.006 24995870 PMC4085634

[B36] Tisdale A. Cutillo C. M. Nathan R. Russo P. Laraway B. Haendel M. (2021). The IDeaS initiative: Pilot study to assess the impact of rare diseases on patients and healthcare systems. Orphanet J. Rare Dis. 16 (1), 429. 10.1186/s13023-021-02061-3 34674728 PMC8532301

[B37] Weeke L. C. Brilstra E. Braun K. P. Zonneveld-Huijssoon E. Salomons G. S. Koeleman B. P. (2017). Punctate white matter lesions in full-term infants with neonatal seizures associated with SLC13A5 mutations. Eur. J. Paediatr. Neurology 21 (2), 396–403. 10.1016/j.ejpn.2016.11.002 27913086

[B38] Whitney R. Choi E. Jones K. C. (2023). The neuroimaging spectrum of SLC13A5 related developmental and epileptic encephalopathy. Seizure 106, 8–13. 10.1016/j.seizure.2023.01.014 36701889

[B39] Willmes D. M. Daniels M. Kurzbach A. Lieske S. Bechmann N. Schumann T. (2021). The longevity gene mIndy (I’m Not Dead, Yet) affects blood pressure through sympathoadrenal mechanisms. JCI Insight 6 (2), e136083. 10.1172/jci.insight.136083 33491666 PMC7934862

[B40] Yang G. Cintina I. Pariser A. Oehrlein E. Sullivan J. Kennedy A. (2022). The national economic burden of rare disease in the United States in 2019. Orphanet J. Rare Dis. 17 (1), 163. 10.1186/s13023-022-02299-5 35414039 PMC9004040

[B41] Yang Q.-Z. Spelbrink E. M. Nye K. L. Hsu E. R. Porter B. E. (2020). Epilepsy and EEG phenotype of SLC13A5 citrate transporter disorder. Child. Neurol. Open 7, 2329048X20931361. 10.1177/2329048X20931361 PMC7281881 32551328

